# New Appliances and Things Medical

**Published:** 1902-05-24

**Authors:** 


### NEW APPLIANCES AND THINGS MEDICAL.

[We shall be glad to receive at our Office, 28 & 29 Southampton Street, Strand, London, W.C., from the manufacturers, spec ens of all new preparations and appliances which may be brought out from time to time.]

### BEEF AND MALT WINE.

(James Murray & Sons, Limited, 34 Great Clyde Street, Glasgow.)

This preparation, which is known as the Armstrong Beef and Malt Wine, is composed of an excellent matured port wine containing beef juice and malt extract; it is thus at once a stimulant and a food combined. The great advantage of a tonic preparation of this kind is that invalids who have a distaste for foods of all kinds can often be induced without much trouble to take a stimulant such as port wine, and in the case of Armstrong's preparation the palatable character of the wine is not destroyed by the addition of two such invaluable food elements as meat juice and malt extract. This nourishing wine is likely to be useful for many patients recovering from exhausting diseases, such as influenza or typhoid fever, and for phthisical cases the combination of a valuable food and a stimulant, such as are contained in the wine, will frequently be found in strict accordance with the therapeutic indications.

### FLOOR POLISH AND FURNITURE CREAM.

(Stephenson Brothers, Limited, Bradford, Yorks)

We have examined a sample of Stephenson's Floor Polish and are of the opinion that it is a preparation which presents the merits of a scientific and skilful manufacture. It has cleansing and purifying properties owing to the presence of an antiseptic of admitted value, and as a polish for parquet floors, linoleums, and oil cloths, it can scarcely be beaten. The furniture cream, which is supplied by the same firm, has equal merits as a skilfully manufactured emulsion. Its composition is such as to produce a brilliant and durable gloss on the surface of polished woods and japanned wares, and a practical trial will convince those who use it that, owing to the rapidity with which it accomplishes its object, it is a thoroughly economical furniture cream to put into the hands of busy servants.

